# pH-Responsive Hyaluronic Acid-Based Mixed Micelles for the Hepatoma-Targeting Delivery of Doxorubicin

**DOI:** 10.3390/ijms17040364

**Published:** 2016-03-30

**Authors:** Jing-Liang Wu, Gui-Xiang Tian, Wen-Jing Yu, Guang-Tao Jia, Tong-Yi Sun, Zhi-Qin Gao

**Affiliations:** School of Bioscience and Technology, Weifang Medical University, Wei Fang 261053, Shandong, China; gxtian2008@163.com (G.-X.T.); yuwjwf@163.com (W.-J.Y.); guangtaojia@163.com (G.-T.J.); swgch@wfmc.edu.cn (T.-Y.S.)

**Keywords:** hepatoma-targeting, glycyrrhetinic acid, hyaluronic acid, micelle

## Abstract

The tumor targetability and stimulus responsivity of drug delivery systems are crucial in cancer diagnosis and treatment. In this study, hepatoma-targeting mixed micelles composed of a hyaluronic acid–glycyrrhetinic acid conjugate and a hyaluronic acid-l-histidine conjugate (HA–GA/HA–His) were prepared through ultrasonic dispersion. The formation and characterization of the mixed micelles were confirmed via ^1^H-NMR, particle size, and ζ potential measurements. The *in vitro* cellular uptake of the micelles was evaluated using human liver carcinoma (HepG2) cells. The antitumor effect of doxorubicin (DOX)-loaded micelles was investigated *in vitro* and *in vivo*. Results indicated that the DOX-loaded HA–GA/HA–His micelles showed a pH-dependent controlled release and were remarkably absorbed by HepG2 cells. Compared with free DOX, the DOX-loaded HA–GA/HA–His micelles showed a higher cytotoxicity to HepG2 cells. Moreover, the micelles effectively inhibited tumor growth in H22 cell-bearing mice. These results suggest that the HA–GA/HA–His mixed micelles are a good candidate for drug delivery in the prevention and treatment of hepatocarcinoma.

## 1. Introduction

Synthetic or natural polymer nanoparticles have attracted increasing attention as drug delivery devices because of their chemical structures, surface functionalities, and particle size [1,2,3]. Given their non-toxicity, biocompatibility, and biodegradability, nano-sized self-aggregates composed of natural polysaccharides, such as chitosan [4,5], hyaluronic acid (HA) [6,7], pullulan [8], and dextran [9] have captured the interest of many researchers. Hydrophobically modified polysaccharides can self-aggregate in aqueous media through intra- and/or intermolecular hydrophobic interactions to form nanoparticles with a hydrophobic core and a hydrophilic shell. These amphiphilic polymers are suitable for encapsulating hydrophobic anticancer drugs, such as doxorubicin (DOX) and paclitaxel. Drug-loaded carriers effectively prolong the circulation time and release of drugs in a sustained manner at their target site through the enhanced permeability and retention effects of such carriers [10]. Moreover, nano-sized carriers modified by targeting moieties, such as folic acid [11], antibodies [12], and various ligands, can bind to specific receptors and be absorbed via receptor-mediated endocytosis, thereby bypassing the recognition of P-glycoprotein [13].

HA is a natural linear, negatively charged polysaccharide. As one of the main hydrophilic components of the extracellular matrix, HA is biocompatible, biodegradable, and present in the extracellular matrix and synovial fluids. HA-binding receptors, such as cluster determinant 44 (CD44) and receptor for HA-mediated motility, are overexpressed on many types of tumors [14,15]. Thus, HA is potent as a targeting moiety in pharmaceutical applications for anticancer therapeutics. Recent studies have found that HA conjugates containing anticancer agents, such as paclitaxel [15,16], DOX [17,18], and siRNA [19,20], exhibit an enhanced targeting ability to the tumor and a higher therapeutic efficacy compared with free anticancer drugs.

Liver cancer is a prevalent cancer with a high mortality. Liver-targeting drug delivery is a highly desirable strategy to improve therapeutic outcomes; this strategy has significantly lower toxic side-effects compared with traditional chemotherapy [21]. In the past decade, many scientists have prepared drug delivery carriers with liver-targeting moieties, such as antibodies [22,23], sugars [24], and various ligands, for the targeted therapy of hepatocellular carcinoma. Glycyrrhetinic acid (GA) receptor-mediated drug delivery systems have attracted growing interest for the treatment of liver cancer because of their lower cost than antibodies [25]. GA surface-modified nanoparticles remarkably improve liver-targeting efficiency and inhibit liver cancer growth [26,27,28].

In our previous study, we prepared and characterized amphiphilic HA polymers as nano-delivery vehicles for DOX [29,30]. Self-assembled nanoparticles based on HA have great capabilities in the solubilization and delivery of hydrophobic drugs to target CD44-overexpressing tumor cells. In this study, we aim to develop hepatoma-targeting mixed micelles by combining two strategies: pH-responsible drug release and GA receptor-mediated targeting of hepatoma cells.

Hepatoma-targeting mixed micelles composed of a HA–glycyrrhetinic acid conjugate (HA–GA) and a HA-l-histidine conjugate (HA–His) were fabricated. The physicochemical characteristics of the micelles were investigated using dynamic light scattering, ζ potential measurements, and transmission electron microscopy (TEM). The cellular uptake of DOX-loaded micelles *in vitro* was monitored using human liver carcinoma (HepG2) cells, and the antitumor effect of the DOX-loaded nanoparticles was studied *in vitro* and *in vivo*.

## 2. Results and Discussion

### 2.1. Synthesis and Characterization of HA-l-Histidine Conjugate (HA–His) and Hyaluronic Acid-Glycyrrhetinic Acid (HA–GA) Conjugates

**Figure 1:**
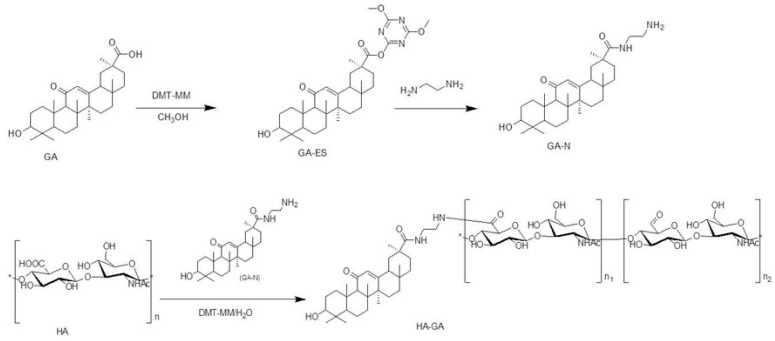
Synthetic route of hyaluronic acid-glycyrrhetinic acid (HA–GA) conjugate via 4-(4,6-dimethoxy-1,3,5-triazin-2-yl)-4-methylmorpholinium chloride (DMT–MM)-mediated coupling reactions.

A HA–His conjugate was synthesized as previously described [30], and a HA–GA conjugate was synthesized using HA as a hydrophilic segment and GA as a hydrophobic segment through 4-(4,6-dimethoxy-1,3,5-triazin-2-yl)-4-methylmorpholinium chloride (DMT–MM)-mediated coupling reactions (Figure 1). 1-Ethyl-3(3-dimethylaminopropyl) carbodiimide/*N*-hydroxysuccinimide (EDC/NHS) is the standard method to activate carboxyl groups for amide formation. However, DMT–MM is more efficient than EDC/NHS in ligating amines to HA and does not require accurate pH control or pH shift during the reaction [31,32].

**Figure 2:**
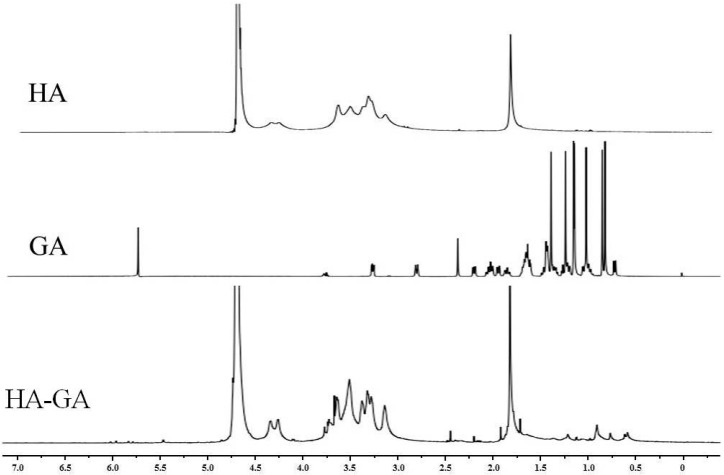
^1^H-NMR spectra of HA, GA, and the HA–GA conjugate.

The structure of the HA–GA conjugate was confirmed via ^1^H-NMR. The ^1^H-NMR spectra of HA, GA, and the HA–GA conjugate were shown in Figure 2. Chemical shifts corresponding to HA (2.0 and 3.3–4.7 ppm) were observed, and the characteristic peaks at 0.64–1.37 ppm were assigned to the typical protons of the GA moiety. The successful introduction of GA into HA was indicated by the presence of characteristic peaks at 0.6–1.4 ppm [27,33]. The degree of substitution (DS) was determined by comparing the average number of GA molecules attached per 100 HA molecules. The DS was controlled within 3–20 by varying the free ratio of GA to HA polymers.

### 2.2. Preparation and Characterization of HA–GA/HA–His Micelles

**Figure 3:**
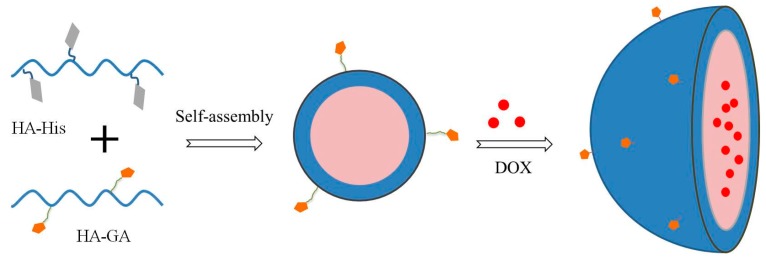
Preparation of HA–GA/HA–His mixed micelles.

Self-assembled HA–GA/HA–His mixed micelles were prepared using a simple ultrasonic method. His and a large part of GA were clustered in the core, whereas the hydrophilic backbone of HA served as the shell, as it is shown in Figure 3.

The average size of the HA–GA/HA–His mixed micelles was influenced by the mixing ratio of the two polymers. As shown in Table 1, the particle size of the mixed micelles increased from 147.5 to 328.2 nm as the ratio of HA–GA to HA–His (*w*/*w*) was decreased from 1:1 to 1:20. This result indicates that the particle size of the mixed micelles increased with increasing HA–His content. The ionization–deionization of the imidazole ring possibly caused the disintegration and aggregation of the micelles in aqueous solution. The HA–GA/HA–His (1:1, *w*/*w*) micelles were chosen as the representative candidate for further studies because of their low particles size.

**Figure 4:**
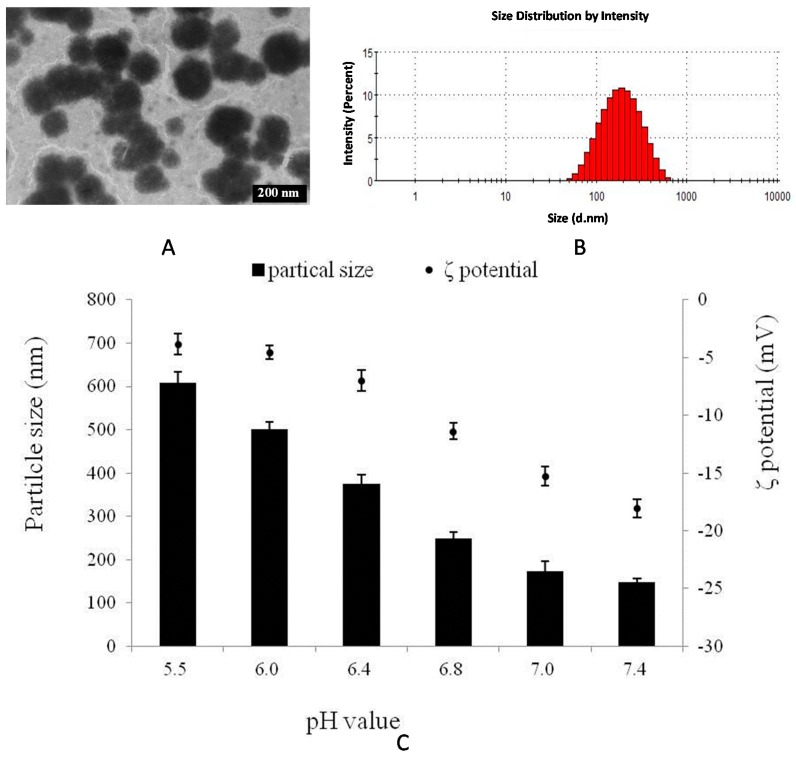
Characterization of HA–GA/HA–His mixed micelles. (**A**) TEM image of mixed micelles; (**B**) Particle size distribution of mixed micelles; (**C**) Particle size and ζ potential of mixed micelles at external pH 7.4–5.5.

The TEM image and particle size distribution of the HA–GA/HA–His micelles in Figure 4 revealed that the micelles were intact, well-separated, and spherical. The pH responsibility of the HA–GA/HA–His micelles was measured to determine the effect of pH on the particle size and ζ potential of the micelles (Figure 4C). The average particle diameter ranged from 147.4 to 158.6 nm at external pH 7.4–7.0. This result suggests that the mixed micelles could be stable at pH value of 7.4 to 7.0. Stepwise shifts in the pH of the solution to lower pH values resulted in a sudden increase in average particle size. The particle diameter increased to 248.5 nm at pH 6.8, whereas increased to 374.2, 500.2 and 607.6 nm at pH 6.4, 6.0 and 5.5, respectively [34]. This pH-sensitive ability was probably due to the deformation of the hydrophobic core induced by the ionizable imidazole rings of His; these rings were protonated at acidic pH, which triggered the dissociation and increased the size of the micelles [35]. The ζ potential was measured to reveal the surface charge variation of the micelles at different pH values. Figure 4C showed that the absolute value of the ζ potential decreased when the pH was changed from 7.4 to 5.5, whereas the HA–His micelles remained negatively charged because of the weak acidity of HA (pI = 2.5). This result may be attributed to the fact that the ionized His group dissociated from the micelle core through the “proton sponge” effect, which decreased the surface charge of the mixed micelles.

### 2.3. Drug Encapsulation Efficiency and in Vitro Drug Release

When the initial feed ratio of DOX to the HA–GA/HA–His micelles was 10 wt %, DOX was physically incorporated into the HA–GA/HA–His mixed conjugate. The drug loading (DL) capacity and encapsulation efficiency (EE) of the DOX-loaded micelles were 91.43% and 8.64%, respectively. This finding indicates that the hydrophobic DOX molecules were efficiently encapsulated into the HA–GA/HA–His micelles in aqueous solution because of the presence of a hydrophobic core in the micelles. The DOX-loaded micelles were found to have a mean diameter of 162 ± 6.8 nm (mean ± SD; *n* = 3). Compared to blank micelles, the DOX-loaded micelles showed a slight increase in micelle size. The surface charge was −17.9 ± 4.7 mV (mean ± SD; *n* = 3) in ζ potential measurement, which showed the high stability in water.

**Figure 5:**
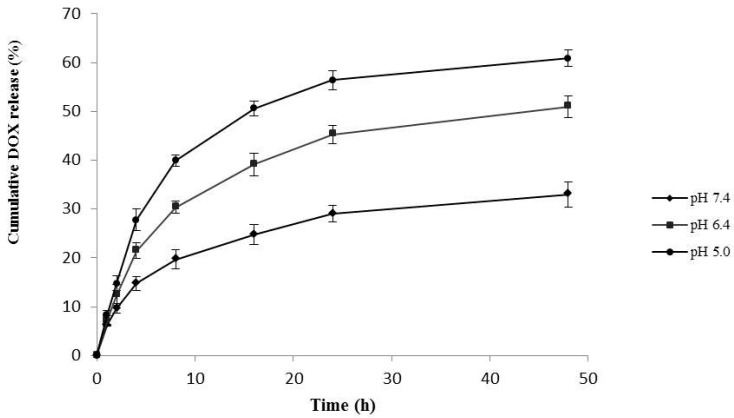
Release behavior of DOX from HA–GA/HA–His micelles at different pH values *in vitro*.

The *in vitro* drug release behavior of the HA–GA/HA–His micelles was investigated under physiological conditions (pH 7.4), tumor acidic microenvironment (pH 6.4), and intralysosomal pH (pH 5.5) at 37 °C. As shown in Figure 5, the DOX release was significantly influenced by pH. Within 5 h, 14.6 wt % of DOX was released from the micelles at pH 7.4, whereas 21.4 and 27.8 wt % were released at pH 6.4 and 5.5, respectively. Within 48 h, 37.9 wt % of DOX was released from the micelles at pH 7.4, whereas 49.5 and 67.9 wt % were released at pH 6.4. The results suggest that the DOX-loaded HA–GA/HA–His micelles exhibited a sustained DOX release under physiological conditions and a faster release under tumor microenvironment and lysosomal pH in tumor cells.

The drug release mechanism of the micelles might be determined by diffusion and/or degradation. The *in vitro* release studies showed that the rate and amount of DOX release increased with decreasing pH. The micelles possibly possessed compact hydrophobic cores composed of His and GA, and the DOX release from the cores was almost completely diffusion controlled under physiological conditions (pH 7.4), which led to a slow release rate. However, the imidazole ring of His was protonated, and the compact hydrophobic cores became swollen at lower pH (6.4 and 5.5) because the charged imidazole ring resulted in large amounts of DOX release [35].

### 2.4. In Vitro Cellular Uptake of GA–HA/His–HA Micelles

**Figure 6:**
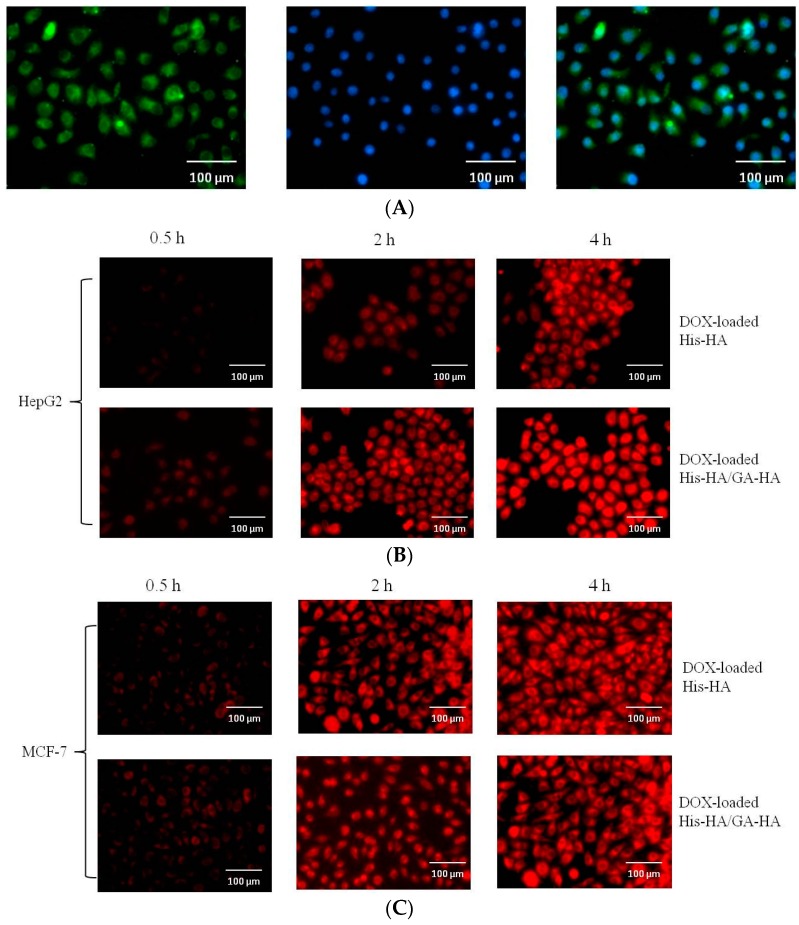
Fluorescence micrographs of HepG2 cells treated with FITC-labeled micelles (**A**); The cellular uptake of DOX-loaded micelles against HepG2 cells (**B**) and MCF-7 cells (**C**). FITC channel for FITC-labeled micelles (green), TRITC channel for DOX (red), and DAPI channel for nucleus (blue) were simultaneously presented.

Fluorescein isothiocyanate (FITC)-labeled micelles were successfully synthesized to investigate the cellular uptake of the HA–GA/HA–His micelles. FITC served as a fluorescence probe to track the HA–GA/His–GA micelles, and cell nuclei were labeled with DAPI (blue fluorescence). As shown in Figure 6A, strong green fluorescent signals were detected in the cytoplasm of HepG2 cells after incubation with FITC-labeled HA–GA/HA–His micelles for 2 h. This result indicates that the micelles were readily internalized into HepG2 cells.

**Figure 7:**
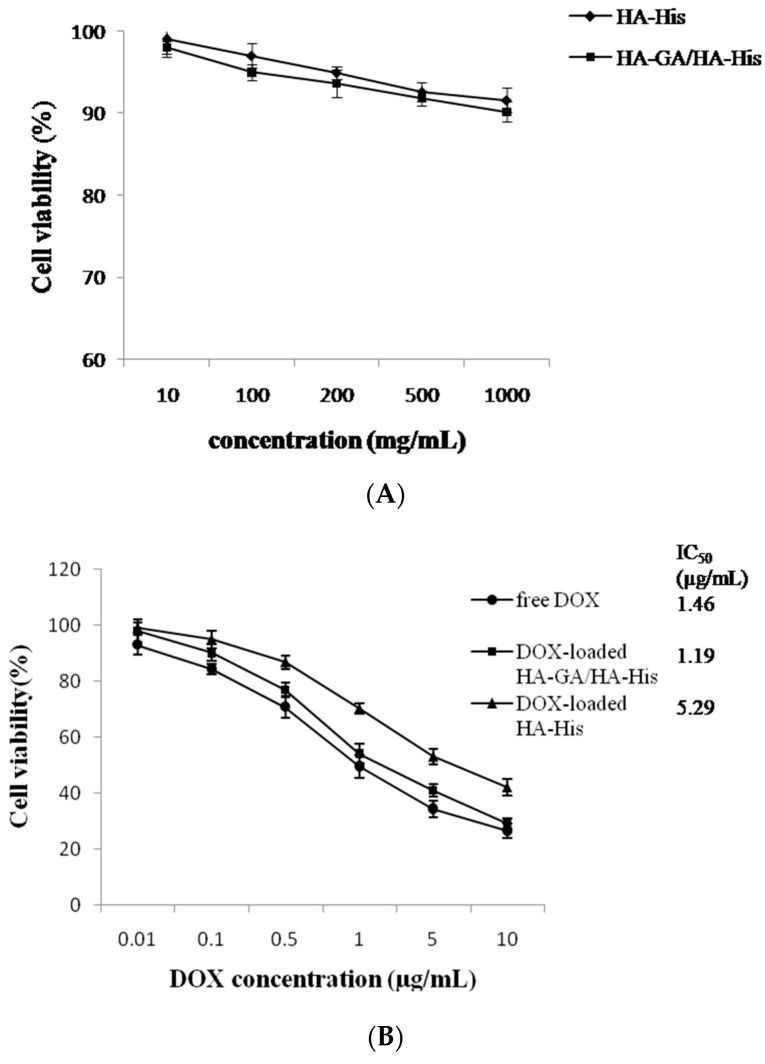
Viability of HepG2 cells after respective treatment with blank micelles (**A**) and free DOX, DOX-loaded HA–His micelles, or DOX-loaded HA–GA/HA–His micelles (**B**) for 48 h.

The presence of GA in HA–GA/HA–His micelles is essential for hepatoma-targeting drug delivery. The intracellular uptake of the DOX-loaded HA–GA/HA–His micelles was investigated using fluorescence microscopy (Figure 7B,C). DOX-loaded micelles were incubated with liver carcinoma cells (HepG2) over-expressing GA-receptor or non-hepatic tumor cells (MCF-7) for 0.5, 2 and 4 h. DOX served as a fluorescence probe to track the DOX-loaded micelles. As shown in Figure 6B, cellular uptake of DOX increased with the extension of incubation time; however there were significant differences between the two DOX-loaded micelles against HepG2 cells. Red fluorescence spots were remarkably observed in the cytoplasm of HepG2 cells after incubation for 2 and 4 h. This finding suggests that the DOX-loaded HA–GA/HA–His micelles were readily taken up by HepG2 cells. By contrast, the red fluorescence of DOX was weak when DOX-loaded HA–His micelles were incubated with HepG2. This phenomenon may be attributed to the fact that GA receptor-mediated endocytosis promoted the cellular uptake of the DOX-loaded HA–GA/HA–His micelles. Moreover, when DOX-loaded micelles were incubated with MCF-7 cells, which did not over-express GA-receptor, no significant difference was observed between two treatments. These results suggest that the presence of GA in the mixed micelles might play a major role in hepatocyte-targeting activity [36,37].

### 2.5. In Vitro Cytotoxicity Assay and in Vivo Anti-Tumor Efficacy

The cytotoxicity against HepG2 cells and the antitumor efficacy in hepatoma-tumor-bearing mice were tested to evaluate the antitumor efficiency of the DOX-loaded HA–GA/HA–His micelles. The cytotoxicity of the blank micelles and DOX-loaded micelles was investigated using the 3-(4,5-dimethylthiazol-2-yl)-2,5-diphenyl tetrazolium bromide (MTT) assay. The cell viability of blank micelles was measured after 48 h incubation. As shown in Figure 7A, the cellular viability of blank micelles was over 90% at all concentrations (10–1000 mg/mL) tested. It indicated that HA–His micelles and HA–GA/HA–His mixed micelles were biocompatible and could be used as nano-sized vehicle for DOX delivery.

The viability of HepG2 cells after respective incubations with free DOX, DOX-loaded HA–GA/His–GA micelles, or DOX-loaded HA–His micelles for 48 h was evaluated. As shown in Figure 7B, the three DOX formulations exhibited similar dose-dependent cytotoxic effects against HepG2 cells. The half maximal inhibitory concentration (IC50) of DOX-loaded HA–GA/HA–His micelles was determined to be 1.46 DOX equiv/mL, which was nearly 4-fold lower than that of DOX-loaded HA–His micelles (5.29 DOX equiv/mL), and comparable to that of free DOX (1.19 µg DOX/mL). These results might be attributed to the fact that the introduction of GA to the micelles promoted the cellular uptake of DOX because of the high affinity of the GA-modified micelles to HepG2 cells, thereby endowing the micelles a remarkable cytotoxicity against the hepatoma cells [21,36].

**Figure 8:**
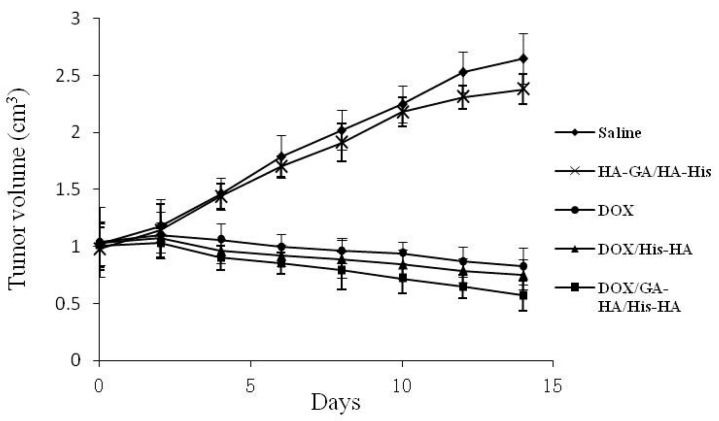
Inhibition of tumor growth by respective injection of physiological saline (control), blank HA–GA/HA–His micelles, free DOX·HCl, DOX-loaded HA–His micelles, or DOX-loaded HA–GA/HA–His micelles. The data represent the mean of the tumor volume from six mice ± SD.

The antitumor efficacy of the DOX-loaded micelles on the H22 cell-bearing mice was tested. As shown in Figure 8, tumor growth was observed for 14 days in the mice injected with free DOX, DOX-loaded HA–His micelles, DOX-loaded HA–GA/HA–His micelles, blank HA–GA/HA–His micelles, or physiological saline (control). For the groups of saline and blank HA–GA/HA–His micelles, the tumors grew rapidly with similar tendency during the test period, indicating the inefficacy of HA–GA/HA–His micelles. Whereas, the tumor growth could be markedly inhibited in the drug-treated groups. *In vivo* tumor inhibition ratio of DOX-loaded HA–GA/HA–His micelles was 78.5%, showing higher than that of DOX-loaded HA–His micelles (69.1%) and free DOX injection (72.3%). Compared to free DOX, the DOX-loaded HA–GA/HA–His micelles possibly increased drug accumulation in the tumor, which improved their antitumor efficiency. Moreover, the result showed that DOX-loaded HA–GA/HA–His micelles exhibited stronger tumor regression than DOX-loaded HA–His micelles. This suggests that the introduction of GA molecular might be effective for hepatoma-targeting delivery, resulting in higher antitumor efficacy than GA-free micelles [38].

**Figure 9:**
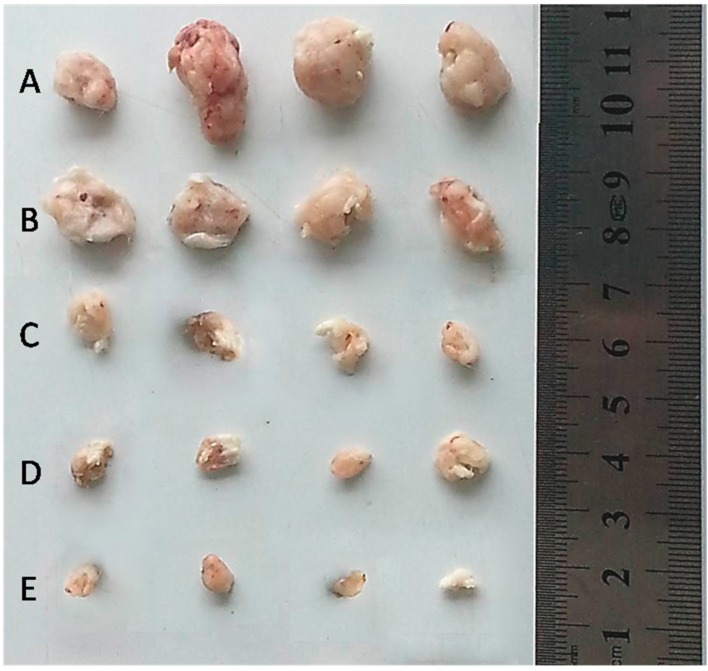
Tumors of each group treated with saline (**A**); black HA–GA/HA–His micelles (**B**); free DOX·HCl (**C**); DOX-loaded HA–His micelles (**D**); and DOX-loaded HA–GA/HA–His micelles (**E**) on the 14th day.

All H22 cell-bearing mice were sacrificed after 14 days of treatment, and the tumors were extracted. As shown in Figure 9, the tumor size of the free DOX·HCl group, or the DOX-loaded micelle group was smaller than that of the saline group. This result indicates the remarkable antitumor effect of the free DOX·HCl and DOX-loaded micelles. Compared with the group treated with the DOX-loaded HA–His micelles, the group treated with the DOX-loaded HA–GA/HA–His micelles had a smaller tumor size. This result suggests that the HA–GA/HA–His mixed micelles possessed a higher targeting capacity than the HA–His micelles because of the active hepatic targeting properties of GA.

The results of cytotoxicity assay *in vitro* and antitumor efficacy experiment *in vivo* showed that the HA–GA/HA–His micelles can deliver DOX effectively and inhibit the growth of hepatocellular carcinoma. Thus, these micelles have potential importance in pharmaceutical applications.

## 3. Materials and Methods

### 3.1. Materials

Sodium hyaluronate (*M*_w_: 70 kDa) was purchased from Shandong Freda Biopharm Co., Ltd. (Ji’nan, China). GA (purity >98% by HPLC) was purchased from Fujie Pharmaceutical Co., Ltd. (Xi’an, China). His was purchased from Sinopharm Chemical Reagent Co., Ltd (Shanghai, China). DOX·HCl was purchased from ShanXi powerdone Pharmaceutical Co., Ltd (Xi’an, China). FITC was purchased from Huasheng Co., Ltd (Zhejiang, China). EDC, NHS, and DMT–MM were purchased from Shanghai Medpep Co., Ltd., (Shanghai, China) and pyrene was purchased from Sigma (St. Louis, MO, USA). All other chemicals were analytical grade.

Human hepatic cell line (HepG2), MCF-7 cells and female BALB/c mice (age: 7 weeks; weight: approximately 20 g; approved by the WFMU Animal Research Ethics Committee, approval No. 302-003, Date: 30/5/2015) were obtained from WeiFang Medical University. Care and handling of the animals were in strict compliance with the “Guide for the Care and Use of Laboratory Animals”. MTT was purchased from Sigma-Aldrich (St. Louis, MO, USA). RPMI-1640 medium and fetal bovine serum were purchased from Beijing Solarbio Co., Ltd. (Beijing, China).

### 3.2. Synthesis of HA–GA and HA–His Polymers

A HA–GA conjugate was synthesized from HA (70 kDa) and GA. GA in methanol was activated with DMT–MM to form active ester. After the rotary evaporation of methanol, the active ester was slowly added to ethylene diamine solution, and the mixture was stirred at room temperature overnight. The diamine-modified GA (GA-N) was purified through column chromatography. The HA–GA conjugate was synthesized by modifying GA-N to the backbone of HA in the presence of DMT–MM. After freeze-drying, the chemical structure of the GA-HA conjugate was determined via ^1^H-NMR (JNMECP-600, JEOL, Tokyo, Japan), for which the sample was prepared by dissolving the conjugate in D_2_O. The routes of synthesis are illustrated in Figure 1.

A HA–His conjugate was synthesized as previously described with some modifications [30]. In brief, HA in formylamine was activated by EDC and NHS (mol_HA_:mol_EDC_:mol_NHS_ = 1:1.2:1.2). Then, His (the molar ratio between the carboxyl group of HA and the amino group of His was 1:6) in *N*,*N*-dimethylformamide (DMF) was slowly added to the HA solution. The reaction mixture was stirred for 24 h at room temperature under nitrogen atmosphere. After filtration, the solution was freeze-dried to obtain the HA–His conjugate.

### 3.3. Preparation and Characterization of HA–GA/HA–His Mixed Micelles

HA–GA/HA–His mixed micelles were prepared by ultrasonic dispersion. In brief, the HA–GA/HA–His conjugates were suspended in phosphate buffered saline (PBS, pH 7.4) under gentle shaking. The solution was sonicated three times using a probe-type sonifier (VCX-750, Sonics & Materials, Newtown, CT, USA) at 90 W, and the pulse was turned on for 2 with 3 s intervals under ice bath.

The particle size of the HA–GA/HA–His micelles was measured by Malvern Zetasizer 3000 HAS (Malvern Instruments Ltd., Malvern, UK). All measurements were conducted at 635 nm and 25 °C with a detection angle of 90°. The concentration of micelles was maintained at 1 mg/mL, and each batch was analyzed in triplicate. The ζ potential of the HA–GA/HA–His micelles was measured using Malvern Zetasizer 3000 HSA. Each sample was measured three times at 25 °C. Each experimental result was an average of three independent measurements.

The morphology of the micelles was observed via TEM (Philips TZOST, Philips Tecnai Co., NED, Amsterdam, Netherlands). In brief, one drop of the HA–GA/HA–His micelle suspension was placed on a copper grid. The grid was allowed to dry at room temperature and was examined under an electron microscope.

### 3.4. Preparation and Characterization of DOX-Loaded Micelles

DOX-loaded HA–GA/HA–His micelles were prepared using a dialysis method as previously described [39]. In brief, DOX·HCl was dissolved in DMF in the presence of triethylamine (1.3 times molar quantity of DOX). HA–GA/HA–His conjugates in formamide were mixed with the DMF solution of DOX. The suspension was stirred overnight using a magnetic stirrer. Subsequently, the system was dialyzed against deionized water to remove the unloaded drugs, DMF, TEA, formamide, and triethylammonium chloride by a dialysis bag (MWCO 7000). The dialysis solution was freeze-dried to obtain DOX-loaded micelles, which were stored at 4 °C.

For DL and EE measurements, 5 mg of the freeze-dried DOX-loaded micelles were dissolved in 10 mL of formamide by gentle heating. The absorbance of DOX was analyzed using a UV–vis spectrophotometer at 480 nm. The concentration of DOX in solution was obtained using the standard curve, and DL and EE were calculated using Equations (1) and (2), respectively. All measurements were performed in triplicate.

DL (%) = *W*s/*W*c × 100% (1)

EE (%) = *W*s/*W*a × 100% (2)

*W*s = amount of DOX in the micelles; *W*c = weight of the micelles after freeze-drying; and *W*a = total amount of the DOX·HCl added.

### 3.5. In Vitro Drug Release Study

The *in vitro* release of DOX from the DOX-loaded micelles was performed using a dialysis method in PBS with different pH values of 7.4, 6.4 and 5.5. In brief, 5 mg of the DOX-loaded micelles were resuspended in 5 mL of PBS. The suspension was transferred to a dialysis bag and then dialyzed against 50 mL of the release medium at 37 °C with stirring at 100 rpm. At predetermined time intervals, 4 mL of medium was removed and replaced with the same amount of fresh release medium. The cumulative amount of DOX released was calculated in accordance with the standard curve by using a UV spectrophotometer at 480 nm [40]. The *in vitro* release experiments were carried out in triplicate at each pH.

### 3.6. In Vitro Cellular Uptake Studies

#### 3.6.1. Cell Culture

HepG2 cells were maintained at 37 °C in a humidified atmosphere of 5% CO_2_ in RPMI-1640 medium containing 10% FBS, 100 U/mL penicillin, and 0.1 mg/mL streptomycin.

#### 3.6.2. *In Vitro* Cellular Uptake of Micelles

HepG2 cells were seeded in six-well plates at a density of 5 × 10^4^ cells/mL at 37 °C. After 24 h of incubation, the cells reached 70%–80% confluence, and the medium was replaced with 2 mL of serum-free culture medium containing FITC-labeled micelles, DOX-loaded HA–His micelles, or DOX-loaded HA–GA/HA–His mixed micelles, respectively, for 2 h at 37 °C. Then, the cells were washed with cold PBS and fixed with 4% paraformaldehyde solution. Finally, the intracellular localization of the FITC-labeled micelles and DOX-loaded micelles was visualized via fluorescence microscopy.

### 3.7. In Vitro Cell Cytotoxicity Assay

The anti-proliferative effect of the DOX-loaded micelles on HepG2 cells was tested using an MTT assay. The cells were seeded in 96-well plates at a density of 5 × 10^3^ cells/well and then incubated for 24 h in an incubator (37 °C, 5% CO_2_). The culture medium was replaced by free DOX HCl, DOX-loaded HA–His micelles, or DOX-loaded HA–GA/HA–His micelles, and the cells were incubated for additional 48 h. Then, 20 mL of MTT solution (5.0 mg/mL) was added to each well, and the cells were incubated for another 4 h at 37 °C. Subsequently, the medium was removed and 200 mL of DMSO was added to dissolve the formazan crystals. Cell viability was calculated at 490 nm using a microplate reader (FLx800B, Bio-Tek, Winooski, VT, USA).

### 3.8. In Vivo Antitumor Efficiency

H22-bearing mice were obtained by subcutaneous inoculation in the flank of BALB/c female mice with 0.1 mL of a cell suspension containing 1 × 10^6^ H22 cells. When the tumors reached about 100 mm^3^, groups comprising six mice were randomly assigned to one of the following treatments: physiological saline (control), blank HA–GA/HA–His micelles, free DOX·HCl, DOX-loaded HA–His micelles, and DOX-loaded HA–GA/HA–His micelles. Drugs were administered in an equivalent volume of 0.2 mL at a dose of 10 mg DOX/kg body weight. The tumor volume was monitored, and the tumor volume was calculated as follows: tumor volume (mm^3^) = width^2^ × length/2. Finally, the mice were sacrificed, and the tumors were dissected. The body weight, viability, and other adverse reactions of the tumor-bearing mice were observed and recorded throughout this study.

### 3.9. Statistical Analysis

All experimental data were expressed as the mean value with standard deviation (mean ± SD). One-way ANOVA was used to compare the differences in the results. Probability values less than 0.05 (*p* < 0.05) were considered to indicate statistical significance.

## 4. Conclusions

Hepatoma-targeted mixed micelles composed of HA–GA and HA–His conjugates were prepared conveniently through ultrasonic dispersion. DOX-loaded mixed micelles demonstrated a pH-sensitive controlled release of DOX. Moreover, the *in vitro* cell uptake results showed that the introduction of GA to the micelles significantly increased the affinity of the micelles to HepG2 cells. In addition, the DOX-loaded HA–GA/HA–His micelles exhibited remarkable cytotoxicity against HepG2 cells and effectively inhibited tumor growth in H22 cell-bearing mice.

## Figures and Tables

**Table 1:** Mean diameters and ζ potential measurements of HA–GA/HA–His mixed micelles at different rates.

HA–GA/HA–His	DLS (nm)	PDI	ζ Potential(mV)
1:1	147.5 ± 11.8	0.151	−18.3 ± 1.2
1:2	182.3 ± 15.1	0.148	−19.1 ± 1.4
1:5	239.1 ± 25.8	0.184	−21.5 ± 0.7
1:10	291.6 ± 31.2	0.216	−22.6 ± 1.3
1:20	328.2 ± 36.7	0.209	−24.2 ± 1.8
0:1	327 ± 47.2	0.289	−24.4 ± 2.4
